# Exploring healthcare facilities’ readiness for standard precautions in infection prevention and control: a cross-country comparative analysis of six low- and middle-income countries using national cross-sectional surveys

**DOI:** 10.7189/jogh.15.04205

**Published:** 2025-07-21

**Authors:** Md Abdullah Al Jubayer Biswas, Scott J Adams, Li Xing, Prosanta Mondal, Michael Szafron

**Affiliations:** 1 Collaborative Biostatistics Program, University of Saskatchewan, Saskatoon, Saskatchewan, Canada; 2 Department of Medical Imaging, University of Saskatchewan, Saskatoon, Saskatchewan, Canada; 3 Department of Mathematics and Statistics, University of Saskatchewan, Saskatoon, Saskatchewan, Canada; 4 Department of Community Health and Epidemiology, University of Saskatchewan, Saskatoon, Saskatchewan, Canada; 5 School of Public Health, University of Saskatchewan, Saskatoon, Saskatchewan, Canada

## Abstract

**Background:**

Despite the significant morbidity and mortality caused by healthcare-associated infections worldwide, especially in low- and middle-income countries (LMICs), there is a lack of understanding of the readiness to apply standard precautions for infection prevention and control (IPC) in healthcare facilities across different LMICs.

**Methods:**

We analysed nationally representative health system data from the Service Provision Assessment surveys for six selected LMICs – Afghanistan, the Democratic Republic of Congo, Haiti, Nepal, Senegal, and Bangladesh. We recorded seven tracer items of standard precautions into binary elements. We calculated a readiness index based on the World Health Organization’s Service Availability and Readiness Assessment manual. We utilised survey-weighted multivariable generalised estimating equations to identify factors associated with the readiness index.

**Results:**

Among 6054 healthcare facilities, 55% (95% confidence interval (CI) = 53.1, 56.5) of necessary standard precautions were available, ranging from 48.1% in the Democratic Republic of the Congo to 65% in Nepal. Readiness varied by service area, with the tuberculosis service area being the least prepared at 38% and the general outpatient service area being the most prepared at 66%. Facilities in Nepal and the urban regions showed higher readiness, with mean (x̄) differences of 16% (95% CI = 13.6, 17.9) and 3% (95% CI = 1.8, 4.9) compared to the Democratic Republic of the Congo and rural areas, respectively.

**Conclusions:**

We revealed significant deficiencies in standard precautions within healthcare facilities across six LMICs, notably in rural areas. The findings underscore an urgent need for targeted interventions to improve IPC strategies, particularly in domains like tuberculosis care.

Healthcare-associated infections (HAIs) significantly contribute to global morbidity and mortality [1]. Literature documents HAIs’ burden and complications in both high-income countries (HICs) and low- and middle-income countries (LMICs). Annually, hundreds of millions of patients suffer from HAIs, disproportionately affecting LMICs [1]. The World Health Organization (WHO) reports significant disparities in HAI prevalence between HICs and LMICs [2]. In HICs, seven out of 100 hospitalised patients acquire an HAI, while in LMICs, this figure is 15 out of 100, with a higher risk of severe outcomes [2]. There are also regional disparities within LMICs [3–7]. For example, HAI prevalence is 8–30% in Bangladesh, 3.31% in Nepal, 17.0% in the Democratic Republic of the Congo, 10.9% in Senegal, and 16.0% in Afghanistan [3–7]. Beyond disease burden, HAIs impose a significant economic burden. In the USA, hospitals spend USD 35.7–45.0 billion annually on direct medical costs related to HAIs, while in Europe, the annual cost is estimated at USD 7.3 billion [8]. Unfortunately, while comprehensive data on HAI-related costs are limited, the economic impact is likely proportionally higher due to resource constraints and higher infection rates [1,8].

In 2017, the WHO introduced a comprehensive, evidence-based infection prevention and control (IPC) strategy to decrease HAIs significantly [9]. Despite the WHO’s recommendations, implementing IPC measures in LMIC settings remains challenging due to unidentified key risk factors [10]. In most LMIC settings, essential practices like maintaining hand hygiene and using facemasks and gloves are sometimes compromised due to the limited availability of basic IPC-related resources [10]. A study of 135 healthcare settings across 39 LMICs and 27 high-income countries found that only 58% of LMIC healthcare facilities had formal IPC programs, compared to 89% in high-income countries [11]. Moreover, LMIC healthcare facilities had more limited access to a reliable water supply for handwashing and antiseptic hand rub [11]. A review of 36 relevant studies also identified a significant hurdle in IPC practices among healthcare workers and managers – insufficient availability and subpar quality of personal protective equipment, including masks, face shields, gloves, and gowns [12].

Standard precautions are essential IPC measures in healthcare, crucial for reducing HAIs and their economic costs by curbing pathogen transmission among healthcare providers, patients, and visitors [13]. Despite their importance, LMIC healthcare facilities severely lack basic standard precaution amenities. A comprehensive 2018 study of 129 557 health facilities across 78 LMICs revealed significant variations in service coverage based on facility type, managing authority, and location [14]. A study of 7948 facilities in multiple LMICs revealed that only 12.7% of facilities in Nepal and 17.0% in the Democratic Republic of the Congo had waste bins [15]. Medical masks were available in just 26.4% of facilities in Tanzania and 30.5% in Nepal [15]. Eye protection was found in only 5.7% of facilities in Bangladesh, 5.4% in Senegal, and 11.0% in the Democratic Republic of Congo [15]. Additionally, only 6.8% of healthcare facilities in Nepal and 19.0% in Bangladesh had standard precaution guidelines [15]. A study by Kanyangarara et al., covering 16 456 facilities across 18 sub-Saharan African countries, found that 26% lacked basic hand hygiene resources like soap, running water, or alcohol-based hand rubs [16]. The deficits of standard precaution readiness in LMICs are also associated with the specific characteristics of healthcare facilities.

Despite numerous studies estimating the availability of basic standard precaution amenities in various LMIC healthcare facilities, to our knowledge, only a few have used the Service Availability and Readiness Assessment (SARA) as a common framework to compare. There is still a lack of understanding regarding LMIC healthcare facilities’ readiness to implement standard precautions. In our previous study in Bangladesh, using SARA, we revealed that only 44% of the necessary elements for standard precautions were available, indicating significant deficiencies in IPC resources such as sharps disposal, hand hygiene facilities, pedal bins, and disinfectants [17]. In these studies, a comprehensive cross-country comparison using a common framework (*i.e.* SARA) was not conducted, which provides an in-depth understanding of shared and unique challenges in IPC implementation across diverse LMIC settings.

Building on our prior research, which was limited to Bangladesh, in our current study, we apply the WHO’s SARA tool across six selected LMICs, which provides a standardised, comparative assessment of standard precaution readiness related to IPC readiness. Unlike prior assessments, in our current multi-country analysis, we aimed to explore the readiness of healthcare facilities to implement standard precautions for infection prevention. Our findings will help identify gaps and country-specific deficiencies hindering IPC implementation in LMIC settings. By providing a comparative perspective, we not only update existing knowledge but also provide a broader perspective on variations in standard precautions readiness, addressing an aspect largely unexplored in the literature. Moreover, by applying the WHO’s SARA tool across these six countries, our study findings allow us to conduct cross-country comparisons. The findings also provide essential baseline information for policymakers to identify gaps and prioritise interventions to improve IPC implementation in resource-limited settings.

## METHODS

### Study data source and procedures

We analysed publicly available health system data from the service provision assessment (SPA) surveys [18–23]. The SPA surveys, designed with input from the WHO, United Nations International Children’s Emergency Fund (UNICEF), and various Ministries of Health, gather essential data on service availability and quality within the health systems of participating countries [18–23]. The surveys were a collaborative effort that ensured that data collection aligned with country-specific healthcare priorities, enhancing the reliability and policy relevance of the data set. The methodology for the SPA survey is detailed on the Demographic and Health Survey (DHS) program website [18–23].

We chose the LMIC countries if the available data met the following criteria: 1) SPA survey data were publicly available for each country, 2) the survey components for each country were adopted from the DHS program, 3) survey data standardisation was performed in consultation with key stakeholders such as each country’s Directorate General of Health Services and Directorate General of Family Planning Services, 4) the survey data set for each country were published in 2019 or later, and 5) the survey was implemented under the direct collaboration of the Ministry of Health of each country. With these criteria, we identified six LMICs – Afghanistan, the Democratic Republic of Congo, Haiti, Nepal, Senegal, and Bangladesh.

For each country, we divided SPA survey questionnaires into facility inventory and healthcare provider components [18–23]. The facility inventory section included three modules assessing service availability: infrastructure elements (such as water, electricity, labour force, medical waste disposal, and essential supplies), diagnostic capabilities, and twelve service-specific readiness domains (such as child health, family planning, antenatal care, and chronic disease management) [18–23]. Likewise, healthcare provider questionnaires collected data on educational backgrounds, skills, and experiences. To collect facility inventory surveys, the presence of the specific amenities was recorded using handheld tablet computers.

For our analysis, we extracted the data from the facility inventory survey on readiness for standard precautions in infection prevention. To identify standard precaution measurement components known as tracer items, we relied on the SARA tools, which were collaboratively developed by the WHO and the United States Agency for International Development [24]. Although SARA uses nine elements, known as tracer items, to measure readiness, we included seven elements for this analysis because data for all nine elements were unavailable across all six countries. In contrast, information on these seven elements was consistently available (**Table 1**). A detailed description of the SARA tool is provided elsewhere [25].

**Table 1:** Distribution of surveyed health care facilities by background characteristics, six selected LMICs*

Items	Unweighted	Weighted
Total	6159 (100.0)	6054 (100.0)
Country		
*Afghanistan*	160 (2.6)	142 (2.3)
*Bangladesh*	1524 (24.7)	1524 (25.2)
*Democratic Republic of Congo*	1412 (22.9)	1380 (22.9)
*Haiti*	1033 (16.8)	1007 (16.6)
*Nepal*	1576 (25.6)	1576 (26.0)
*Senegal*	454 (7.4)	425 (7.0)
Location		
*Urban*	2623 (42.6)	2120 (35.0)
*Rural*	3508 (57.0)	3934 (65.0)
*Missing*	28 (0.4)	0 (0.0)
Managing authority		
*Government*	4175 (67.8)	4373 (72.2)
*Local government*	365 (5.9)	304 (5.0)
*Non-governmental organisation*	968 (15.7)	895 (14.8)
*Private for-profit*	651 (10.6)	482 (8.0)

*Presented as n (%).

### Sample size and sampling technique

**Figure 1:**
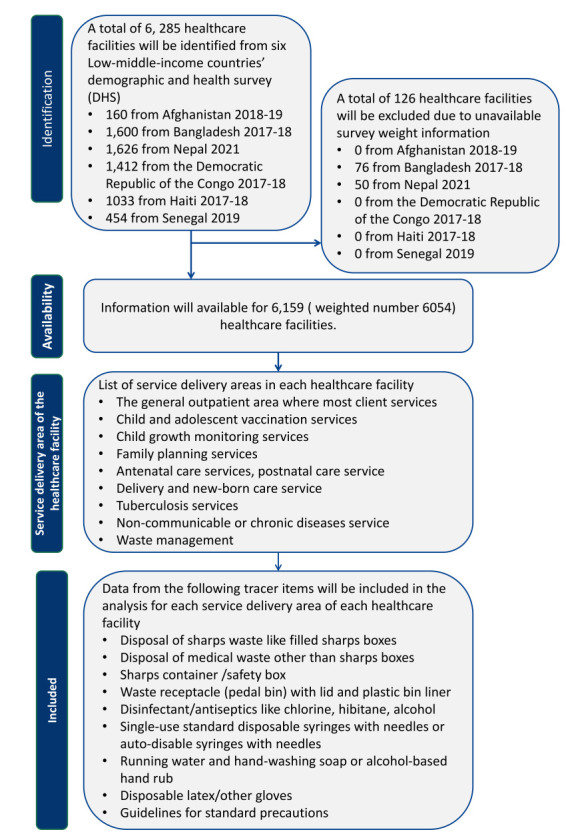
Data extraction flowchart based on the most recent Service Provision Assessment Survey data from six selected countries.

The SPA survey determined sample sizes with specified accuracy across six countries. In Bangladesh, 1600 healthcare facilities were sampled from 19 811 using stratified random sampling by facility type within each division [18]. Nepal’s survey sampled 1626 out of 7598 facilities using stratified random sampling with equal probability systematic sampling by facility type within provinces [18–23]. Afghanistan’s survey included almost all public and private facilities, totalling 160, except in Kabul, where 26 public and 20 private facilities were selected based on administrative factors [18–23]. The Democratic Republic of Congo’s survey sampled 1412 facilities from 12 050 listed by the Ministry of Public Health, excluding health posts, with approximately 50 facilities sampled per province using probability sampling [18–23]. Haiti’s and Senegal’s surveys included all 1033 and 454 healthcare facilities, respectively [18–23]. Furthermore, detailed descriptions of the sampling techniques and management structures for each type of healthcare facility are provided in the survey reports of each country [18–23]. However, for our study, we extracted facility inventory data from 6159 healthcare facilities, excluding 76 in Bangladesh and 50 in Nepal, due to unavailable survey weight information from closures or non-operation during the survey period (**Figure 1**).

### Dependent variables

We extracted data on standard precaution-related variables (tracer items) from the DHS SPA survey, following SARA manual guidelines [24]. After that, we recorded seven tracer items as binary variables, where each tracer item was coded as yes (1) or no (0) [24] (**Table 1**). We used these items to calculate readiness scores according to various service delivery areas of healthcare facilities (**Figure 1**). Additionally, we computed waste management scores using sharps disposal and non-sharps disposal. However, to allow cross-country comparability, we excluded guidelines for standard precautions and availability of storage for infectious waste from the analysis, as these items were not collected in some countries. We determined the percentage readiness scores for each service delivery area, computed by averaging the availability of relevant tracer items and multiplying the result by 100 according to SARA guidelines across each service domain [17,24,26]. The percentage score reflected the average availability of key tracer items for standard precautions in each service delivery area [17,24,26]. Additionally, the overall percentage score for each healthcare facility was then calculated by taking the average of all area-specific readiness scores [17,24,26]. This overall percentage score provided an average measure of the accessibility of tracer items required in all service delivery domains, ranging from 0–100% [17,24,26]. A score of 100% indicated full availability of necessary tracer items in all nine service areas, suggesting optimal IPC readiness [17,26]. Conversely, a score of 0% indicated no availability of essential tracer items, representing a complete lack of readiness for standard precautions [17,26]. We calculated the readiness index using the available data points for each binary tracer item.

### Explanatory variables

We calculated our outcome standard precaution readiness score and examined its association with several explanatory variables, including location and managing authority, selected based on a comprehensive literature review [17,26]. Location was included due to documented disparities between urban and rural healthcare facilities affecting resources, infrastructure, and standard precautions readiness [17,26]. Managing authority was considered for its significant impact on operational policies, resource allocation, and staff training in infection prevention and control. We excluded factors like facility type to avoid complexities in cross-country comparisons due to varied healthcare system structures. Personnel-level factors were also excluded due to data limitations (Table S1 in the **Online Supplementary Document**).

### Statistical analysis

We used descriptive statistics to summarise the characteristics of healthcare facilities in our study and present the standard precaution readiness index scores across explanatory variables. Depending on the variable distribution, we used frequency counts, percentages, x̄, standard deviations (SD), bar graphs, and 95% confidence intervals (CI). Given our aims of estimating country-specific, location-specific readiness (population-averaged effect) rather than healthcare facility-specific readiness, we employed a generalised estimating equations (GEE) technique with a robust sandwich estimator to account for intra-cluster correlation [27]. GEE is commonly used for longitudinal data when repeated measures exist; however, it is also well-suited for cross-sectional clustered data, such as ours, where observations within clusters (countries) are correlated and we are interested in population-level, not individual-level effects [28].

Before performing GEE, we checked key assumptions. The Durbin-Watson statistic (D = 1.6) indicated a positive correlation among readiness index values, suggesting non-independence of observations (Figure S1 in the **Online Supplementary Document**) [29]. We identified an ‘exchangeable’ correlation structure for our multivariable analysis based on the quasi-likelihood under the independence model criterion (Figure S2 in the Online **Supplementary Document**). Third, the intraclass correlation of 1.0 indicated a high similarity within countries, requiring adjustment for clustering (Figure S3 in the **Online Supplementary Document**) [30]. Fourthly, variance inflation factors <5 showed no significant multicollinearity issues (Figure S4 in the **Online Supplementary Document**) [30]. Based on these assumptions, we conducted multivariable GEE with a standard link function and exchangeable correlation structure. We assessed the performance of the GEE model using the quasi-likelihood under the independence model criterion. The results were presented as x̄ differences along with their 95% CIs. We conducted all the analyses adjusted using cluster weight and excluded missing values. We used a 5% significance level. We used SAS, version 9.4 (SAS Institute Inc., Cary, North Carolina, USA) to conduct all analyses.

## RESULTS

### Background characteristics of surveyed healthcare facilities

We included data from 6054 healthcare facilities (unweighted total = 6159). Afghanistan accounted for 142 healthcare facilities (2%), Bangladesh for 1524 (25%), the Democratic Republic of the Congo for 1380 (23%), Haiti for 1007 (17%), Nepal for 1576 (26%), and Senegal for 425 (7%) facilities. Among all the healthcare facilities, approximately two-thirds (65%) of these facilities were located in rural areas, with the majority (72%) managed by government authorities (**Table 1**).

### Tracer items related to the standard precaution readiness index

**Figure 2:**
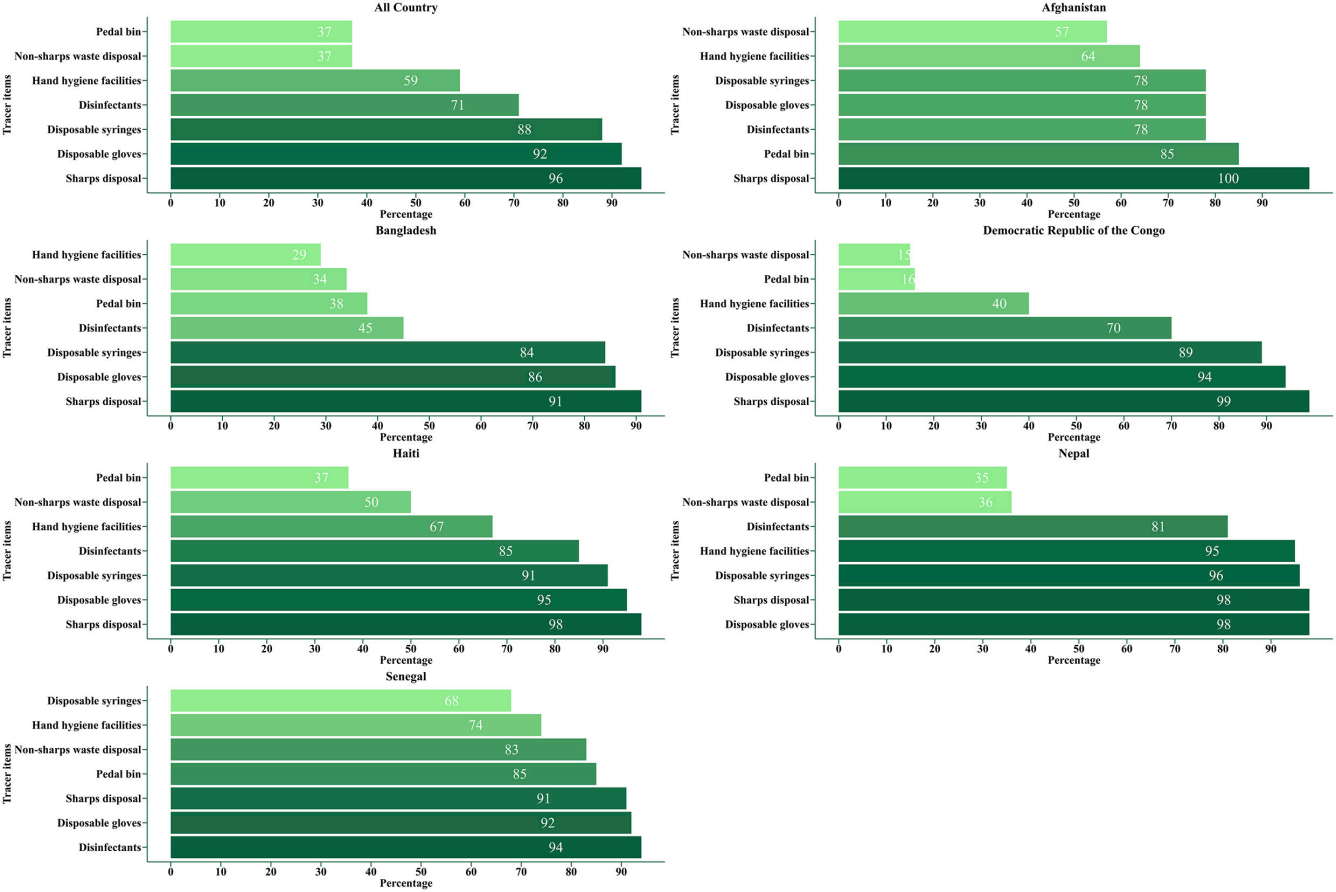
Availability of tracer items related to the standard precaution readiness index according to country.

About two-fifths of healthcare facilities had waste receptacles (pedal bins) (37%), disposal provisions for non-sharp medical waste (37%), and guidelines for standard precautions (27%). Nearly all facilities had systems for sharps waste disposal (96%), disposable gloves (93%), and single-use or auto-disable syringes with needles (89%). Afghanistan and Senegal had the highest availability of pedal bins (85%), while the Democratic Republic of the Congo had the lowest (16%). Disinfectants were most common in Senegal (94%) but less common in Bangladesh (45%). Nepal had the highest availability of disposable syringes (96%) and hand hygiene facilities (95%), whereas Bangladesh had the lowest (29%). For non-sharp waste disposal, Senegal had higher availability (83%), while the Democratic Republic of the Congo had the lowest (15%) (**Figure 2**).

### Standard precautions readiness index according to background characteristics

**Figure 3:**
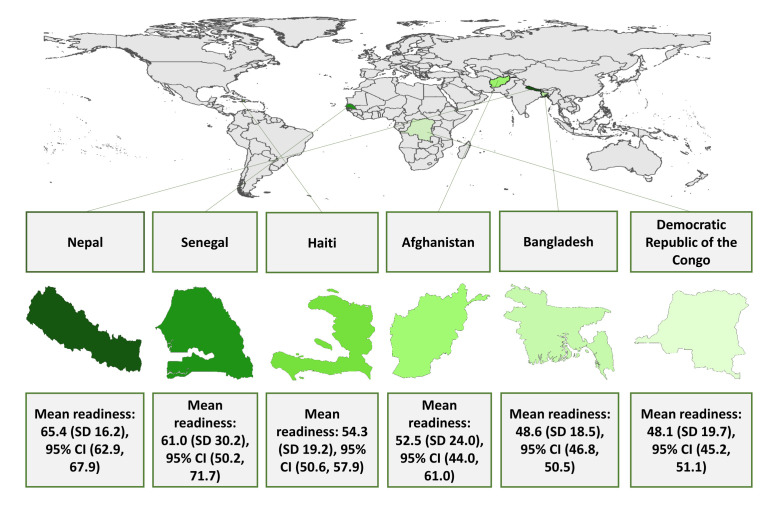
Standard precaution readiness index for six selected LMICs.

In our analysis of 6054 healthcare facilities, on average, just more than half (55%, 95% CI = 53.1, 56.5) had the necessary tracer items for standard precautions for infection control available across nine service delivery areas of our six selected LMICs (**Table 2**). Within the service areas of the healthcare facilities, the availability of essential tracer items for standard precautions varied notably. In the tuberculosis care and delivery and newborn care areas, only around 38% of these items were available on average. In contrast, the general outpatient care services areas showed a higher availability, with 66% of the necessary tracer items present. Additionally, our analysis revealed substantial differences in readiness across countries. In the Democratic Republic of Congo, the readiness index was lower, with only 48% of these items available on average. In contrast, Nepal showed a higher readiness index, indicating that 65% of these tracer items were available on average (**Figure 3**). We also found notable differences in the readiness index according to the managing authority. The readiness index was higher among healthcare facilities managed by government authorities, with 56% of these items available on average. In contrast, non-government authority-managed healthcare facilities showed a lower readiness index, with 50% of these tracer items available on average (**Table 2**).

**Table 2:** Standard precaution readiness index of the healthcare facility according to different characteristics of the healthcare facilities, six selected LMICs

Characteristics of the healthcare facilities	Standard precaution readiness index (%)	
	**x̄ (SD)**	**95% CI**
Among all facilities	54.8 (20.8)	(53.1, 56.5)
Service area of the healthcare facility		
*General outpatient care services*	66.3 (25.4)	(64.4, 68.2)
*Child and adolescent vaccination services*	52.0 (34.3)	(49.2, 54.7)
*Child growth monitoring services*	63.3 (27.4)	(61.4, 65.1)
*Family planning services*	55.7 (33.9)	(53.1, 58.4)
*Antenatal care services, postnatal care services*	62.1 (28.6)	(60.2, 64.0)
*Delivery and newborn care service*	37.6 (39.8)	(33.8, 41.4)
*Tuberculosis care services*	38.5 (38.6)	(35.3, 41.7)
*Non-communicable or chronic disease care service*	55.1 (32.4)	(52.3, 57.9)
*Waste management*	62.4 (27.9)	(60.8, 63.9)
Location		
*Urban*	59.3 (23.9)	(55.9, 62.7)
*Rural*	52.3 (18.7)	(50.8, 53.9)
Managing authority		
*Government*	56.2 (20.6)	(54.1, 58.0)
*Local government*	51.2 (21.0)	(46.8, 55.3)
*Non-governmental organisation*	49.9 (19.9)	(45.7, 54.1)
*Private for-profit*	54.8 (21.1)	(52.1, 57.5)

CI – confidence interval, SD – standard deviation, x̄ – mean

### Associated factors with the standard precautions readiness index

In the unadjusted model, significant associations were found between the readiness index and the location, managing authority, and country of the healthcare facilities. In the adjusted model, we found that urban healthcare facilities had a standard precaution readiness index significantly higher than rural facilities by 5% (95% CI = 4.5, 5.1) (**Table 3**). Similarly, facilities managed by private authorities and government authorities also had higher readiness indexes than those managed by non-profit organisations, with estimated x̄ differences of 5% (95% CI = 4.2, 6.5) and 9% (95% CI = 7.4, 10.2), respectively. A similar higher estimated mean difference of the standard precaution readiness index was found for Haiti (estimated x̄ difference = 6%; 95% CI = 4.8, 6.6), Nepal (estimated x̄ difference = 5%; 95% CI = 4.1, 5.1), and Senegal (estimated x̄ difference = 11%; 95% CI = 8.0, 13.0) compared to Bangladesh.

**Table 3:** Univariable and multivariable generalised estimating equations to investigate associated factors with the standard precautions readiness index, six selected LMICs*

Characteristics of the healthcare facilities	Unadjusted model		Adjusted model	
	**Estimated x̄ difference (95% CI)**	***P*-value**	**Estimated x̄ difference (95% CI)**	***P*-value**
Location				
*Urban*	4.6 (3.8, 5.3)	<0.0001	3.4(1.8, 4.9)	<0.0001
*Rural*	Ref.		Ref.	
Country				
*Afghanistan*	0.08 (1.5, 1.7)	0.920	6.6 (0.5, 12.7)	<0.0001
*Bangladesh*	0.2 (–1.2, 1.6)	0.778	0.2 (–2.0, 2.4)	0.833
*Haiti*	5.7 (4.8, 6.6)	<0.0001	7.4 (5.4, 9.5)	<0.0001
*Nepal*	4.6 (4.1, 5.1)	<0.0001	15.7 (13.6, 17.9)	<0.0001
*Senegal*	10.5 (8.0, 13.0)	<0.0001	11.9 (7.3, 16.5)	<0.0001
*The Democratic Republic of Congo*	Ref.		Ref.	
Managing authority				
*Government*	5.3 (4.2, 6.5)	<0.0001	7.1 (4.5, 9.6)	<0.0001
*Local government*	4.2 (–1.5, 6.9)	0.003	0.3 (–3.1, 3.7)	0.865
*Private for-profit*	8.8 (7.4, 10.2)	<0.0001	9.3 (6.4, 12.1)	<0.0001
*Non-governmental organisation*	Ref.		Ref.	

CI – confidence interval, Ref – reference, x̄ – mean

*Multivariable model adjusted for location, division, and managing authority. GEE fit criteria, QIC = 6087.2.

## DISCUSSION

In our study of standard precaution readiness for infection control in six LMICs healthcare facilities, we revealed deficiencies, with <60% of the essential infrastructure and amenities available. Such inadequate readiness hampers effective IPC measures, posing a significant risk for HAIs and antimicrobial resistance, which can result in increased morbidity, mortality, hospital stays, and healthcare costs, further straining limited resources [1]. Our study findings highlight the need for national IPC strategies to ensure the availability of standard precaution tracer items in key service areas, particularly in rural healthcare facilities, to mitigate nosocomial transmission risks. Targeted investments in healthcare infrastructure and equipment, informed by successful models like Nepal, are crucial for improving preparedness in countries with lower standards, such as the Democratic Republic of the Congo and Bangladesh. International aid may play a vital role in addressing these fundamental gaps. Our findings also provide a baseline for monitoring progress and emphasise the urgent need for targeted interventions to strengthen infection control in resource-limited healthcare settings.

Our findings, indicating readiness levels exceeding three-fifths in Nepal and falling slightly below half in Bangladesh, were comparable with local studies in these countries, such as Nepal (59%) and Bangladesh (44%) [17,26]. Our findings on the proportions of the availability of standard precaution amenities were also consistent with a study that assessed similar proportions in 7948 health facilities across eight LMICs using national health system surveys [15]. However, it was important to note that these studies did not specifically examine standard precautions readiness following SARA guidelines in Afghanistan, the Democratic Republic of Congo, and Haiti, where such analysis was vital for assessing healthcare facility preparedness in these countries regarding IPC.

Our study shows that tuberculosis and delivery/newborn care service areas are most unprepared, with 38% of essential tracer items for standard precautions unavailable across selected countries and service delivery areas. Such shortages may heighten nosocomial transmission risks, especially for pregnant women and newborns, due to their compromised immunity [31]. A scoping review of 77 studies on tuberculosis infection prevention and control in LMICs revealed similar deficiencies in tuberculosis infection prevention and control implementation and essential standard precaution amenities [32]. Another study using a modified Delphi process identified similar infrastructure and supply shortfalls in neonatal service areas [33]. These studies advocate for implementing IPC strategies in tuberculosis, delivery, and newborn care areas, especially in resource-limited settings [32,33]. Our findings also support the need for national IPC strategies to ensure adequate tracer items in these critical service areas.

Our multivariable analysis revealed that healthcare facilities located in the selected LMIC urban areas were significantly more prepared than rural facilities. Our lower standard precaution readiness finding is consistent with previous research. A study by Adhikari et al. reported low standard precaution readiness in rural Nepal healthcare facilities, while Jubayer Biswas et al. reported lower standard precaution readiness in rural Bangladesh healthcare facilities [17,26]. The disparity in standard precaution readiness between rural and urban healthcare facilities could be due to logistic challenges, poor infrastructure, and insufficient funding to improve these conditions [17,26,34–36]. Notably, over 60% of the population in these LMICs lives in rural areas, suggesting that lower readiness in rural facilities could significantly heighten nosocomial transmission risks. To reduce such risk, our study highlights the need to ensure an adequate supply of standard precaution items in rural healthcare facilities in the six selected countries.

Our multivariable analysis revealed that Nepal showed the highest estimated mean difference (highest preparedness) compared to the Democratic Republic of the Congo, likely due to Nepal’s service-oriented policies aimed at strengthening health infrastructure and expanding access to quality care [37]. Similarly, Senegal and Haiti also show significantly higher preparedness, attributed to healthcare reforms and substantial international aid bolstering their infrastructure and supply chain [38–40]. In Senegal, the National Program for Combating Healthcare-Associated Infections (PRONALIN), launched in 2004, may have played a crucial role in strengthening IPC implementation, practices, and preparedness [41]. A study by Binyane et al. reported that PRONALIN improved IPC within the country and served as a model for neighbouring nations, influencing similar programs and advancing regional healthcare standards [41]. Similarly, Haiti’s higher preparedness may be attributed to efforts for healthcare quality improvement after the establishment of the Centres for Disease Control and Prevention (CDC) in 2002 and some governmental quality improvement initiatives like the President’s Emergency Plan for AIDS Relief in 2007, enhancing infection control and healthcare quality [42,43]. Later, after the devastating earthquake on 12 January 2010, international medical teams and humanitarian organisations provided crucial assistance, strengthening the Haitian healthcare system and ensuring a more stable supply of essential medical resources [42,43]. In contrast, the Democratic Republic of the Congo faces low IPC preparedness, which may be due to limited medical supplies, weak infrastructure, political instability, and community mistrust, all of which hinder effective healthcare system management [44]. Despite these challenges, in collaboration with the CDC, the Democratic Republic of the Congo Ministry of Health has strengthened IPC standards in healthcare facilities, particularly in response to COVID-19, which were not tracked in our study [45,46]. Although showing a positive mean difference in preparedness, Afghanistan lags slightly behind, potentially reflecting the impact of foreign aid following conflict; however, its relatively lower preparedness suggests ongoing challenges in fully developing its healthcare system [47,48]. In contrast, Bangladesh’s non-significant difference from the Democratic Republic of the Congo is surprising, indicating similar levels of standard precaution preparedness despite recent progress in health metrics, such as maternal and child mortality [49,50]. In light of the above findings, our study suggests investing in healthcare infrastructure and equipment to improve preparedness in countries like the Democratic Republic of the Congo and Bangladesh, with Nepal’s strategies serving as models and international aid focusing on regions needing basic infrastructure.

Moreover, we found that across all six selected LMICs, healthcare facilities managed by government and private for-profit authorities were significantly more prepared than those managed by non-governmental organisations (NGOs), with government-managed facilities showing a 7% higher readiness score and private for-profit facilities exhibiting a 9% higher readiness score. The low readiness among NGO-operated healthcare facilities may be due to lower access to resources, funding, and infrastructure than typically available to government and private for-profit authority-level facilities [17,26,34,36]. For instance, in Senegal, NGO-run hospitals and clinics play a crucial role in maternal and child healthcare and help bridge healthcare gaps. However, fragmented coordination with multilateral organisations and management challenges may lead to inefficiencies in service delivery and resource utilisation [51]. Similarly, in the Democratic Republic of the Congo, strong oversight and a steady supply of medical essentials improve facility utilisation, yet financial constraints persist. Donated supplies and direct funding are needed to ensure continuous service delivery and better infection control. These disparities are examples of the systemic challenges NGO-operated facilities face in achieving the same level of preparedness as their government and private counterparts.

Our study has significant strengths. It presents infection prevention readiness scores across six countries based on the WHO-prepared SARA guideline, ensuring standardised and robust comparisons across different healthcare settings. It also identifies disparities in readiness by country, facility type, location, and managing authority, providing actionable insights for policymakers to address resource allocation shortages, enhance accountability, and improve evidence-based IPC programs. However, there are notable limitations. First, the study's generalizability is restricted to the six selected LMICs, as it relies on SPA data from these countries. Second, the findings might be outdated due to varying data collection timings across countries, potentially misrepresenting current healthcare conditions. As SPA surveys are periodic, there is a time lag in data collection. While we used the latest available data, it may not reflect post-COVID-19 changes in IPC preparedness. Third, some data points at the service delivery area level were missing and thus excluded from the analysis. Fourthly, potential unmeasured confounders, such as variations in healthcare regulations, workforce training, and facility management practices, could influence our findings. While GEE helps account for clustering within countries, unmeasured confounding bias – the influence of unmeasured variables that may affect the results – cannot be entirely eliminated. Fifthly, many LMICs rely on informal or unregistered healthcare providers who may provide care at facilities not included in SPA surveys, potentially leading to an overestimation of the level of preparedness compared to real-world healthcare conditions.

## CONCLUSIONS

In our analysis, we showed that healthcare facilities in six selected LMICs lack essential resources for effective IPC to reduce HAIs, particularly in rural areas and facilities managed by NGOs. The most significant shortages are in tuberculosis treatment areas, delivery, and newborn care services. These findings can guide policymakers and stakeholders in allocating resources effectively, considering location and management-specific needs. Designing evidence-based interventions that address contextual factors and ensure accountability and sustainability in IPC efforts will better position facilities to reduce nosocomial transmission. Optimal resource allocation and targeted interventions will not only lower infection risks within healthcare settings but also enhance overall readiness for emerging health threats.

## Additional material


Online Supplementary Document
