# Supplementary material for: Exploring healthcare facilities’ readiness for standard precautions in infection prevention and control: a cross-country comparative analysis of six low- and middle-income countries using national cross-sectional surveys
Source: J Glob Health. 2025 Jul 21;15:04205. doi: 10.7189/jogh.15.04205 (PMC12278887; doi:10.7189/jogh.15.04205)
Supplement: Online Supplementary Document [file jogh-15-04205-s001.pdf]

Supplement to: Jubayer Biswas MAA, Adams SJ, Xing L, Mondal P, Szafron M. Exploring healthcare facilities' readiness for standard precautions in infection prevention and control: a cross-country comparative analysis of six low- and middle-income countries using national cross-sectional surveys. J Glob Health. 2025;15:04205.

Table S1. Description of tracer items to measure the standard precaution readiness index

Tracer items and variables	Explanation
Sharps disposal represents the availability of the disposal of sharps waste, such as filled sharps containers that store used needles, syringes, and other sharp medical instruments.	This composite variable captured various sharps waste management methods, such as incineration, open burning, dumping without burning, offsite removal, and the absence of sharps waste disposal. It was categorized into two groups: '1: yes' for active disposal methods (incineration, open burning, dumping without burning, or offsite removal) and '0: no' for no disposal (either never having sharps waste or using other methods).
Non-sharp disposal represented the availability of medical waste that did not include sharps containers, such as used gloves, bandages, and other non-sharp medical materials.	This dichotomous variable recorded medical waste disposal other than sharps boxes in a similar way to sharps waste disposal.
Pedal bins represented the availability of waste receptacles equipped with a foot-operated lid and lined with a plastic bin liner for hygiene.	The presence of waste containers in all care delivery areas of a healthcare facility was categorised as '1: yes' for containers that were either 'observed' or 'reported not seen' and '0: no' for containers that were 'not available.'

Disinfectants represented the availability of cleaning materials, such as chlorine or alcohol-based solutions, used to eliminate or reduce harmful microorganisms on surfaces and equipment.	Availability was also recorded as a dichotomous variable: ‘1: yes’ if the disinfectant was observed or reported as not seen and ‘0: no’ if it was unavailable.
Disposable syringes represented the availability of single-use standard syringes with needles or auto-disable syringes with needles, designed for safe, one-time use.	The presence of auto-disable syringes with needles in all care delivery areas of a healthcare facility was categorised as ‘1: yes’ if syringes were observed or reported as not seen and ‘0: no’ if they were unavailable.
Hand hygiene facilities represented the availability of the provision of running water and hand-washing soap or alcohol-based hand rub to ensure proper hand sanitation.	The presence of running water, hand-washing soap, and alcohol-based hand rub in all healthcare facility service delivery areas was recorded as ‘1: yes’ if these items were observed or reported as not seen and ‘0: no’ if they were unavailable.
Disposable gloves represented the availability of single-use gloves made from latex or other materials designed for protection during medical procedures.	This composite variable indicated the availability of disposable latex gloves. It was classified as ‘1: yes’ if the gloves were observed or reported as not seen, and ‘0: no’ if they were unavailable.
Types of healthcare facility locations	Healthcare facility locations: 1: urban and 2: rural areas.
Managing authority	Healthcare facility ownership was categorised by DHS as follows: government , local government NGOs, and private for-profit entities.
Country	Afghanistan, Democratic Republic of the Congo, Haiti, Nepal, Senegal, and Bangladesh.

DHS = Demographic and Health Survey program; Government indicates the hospital was authorized by the Ministry of Health of the respective country; Local government indicates the hospital was under the jurisdiction of the respective country's local government division; NGO = non-governmental organization.

Assumption Checking

Assumption 1: Independence of the outcome variable values

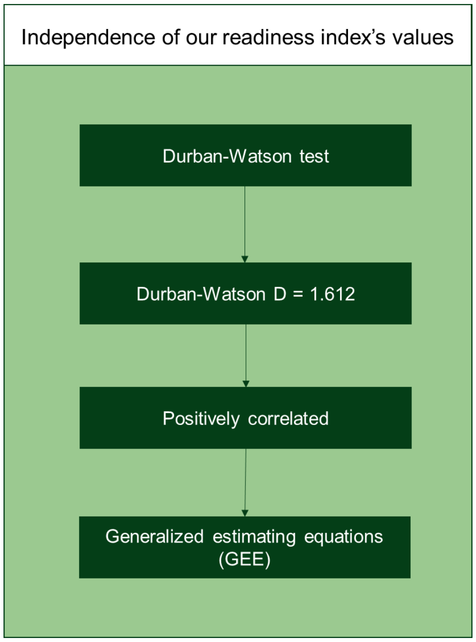

Figure S1: Independence of the outcome variable values. The Durbin-Watson statistic (D = 1.612) suggests the presence of a positive correlation among observations. Given that the dataset includes 6,159 observations with cross-sectional clustered data, a standard regression model assuming independence may lead to misleading standard error. Generalized Estimating Equations (GEE) were chosen as they provide robust standard errors and account for intra-cluster correlation using a working correlation structure [1].

Assumption 2: Correlation structure

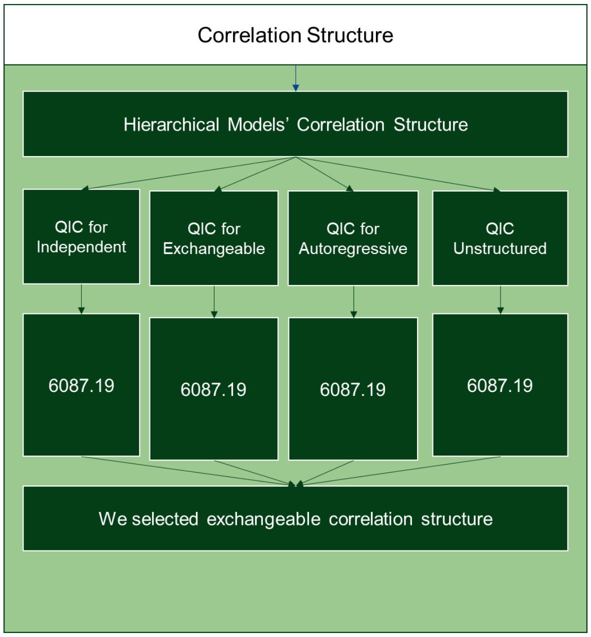

Figure S2: Working correlation structure. Our model was evaluated using multiple working correlation structures, including exchangeable, independent, and unstructured, showing identical QIC values. Identical QIC values indicate that model fit and efficiency remained consistent regardless of the assumed correlation structure [2,3]. Given this, the Exchangeable correlation structure was selected as it assumes an equivalent correlation among observations within the same cluster[2,3].

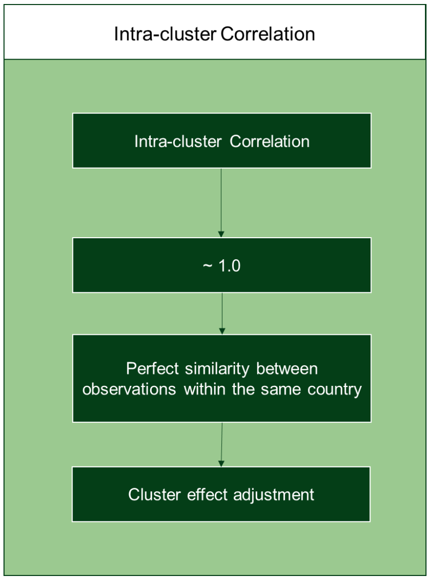

Figure S3: Intraclass correlation. The intraclass correlation (ICC) of 1.0 indicated a high similarity within countries, requiring adjustment for clustering [4,5].

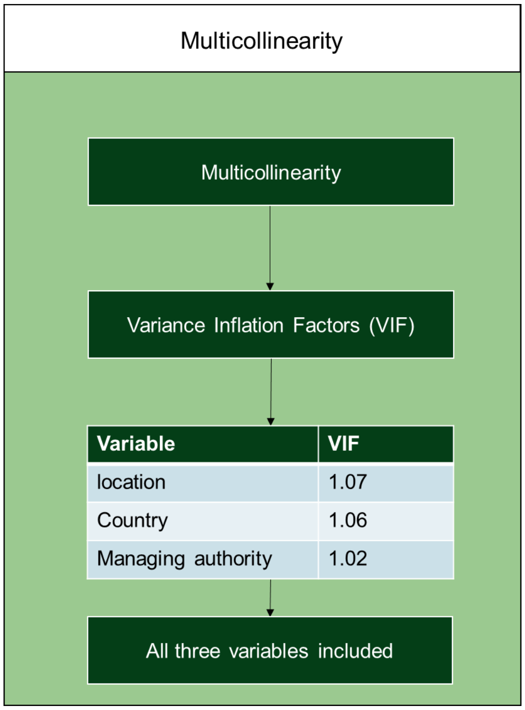

Figure S4: Multicollinearity. VIF values for location (1.07), country (1.06), and managing authority (1.02) indicate minimal multicollinearity among the independent variables.

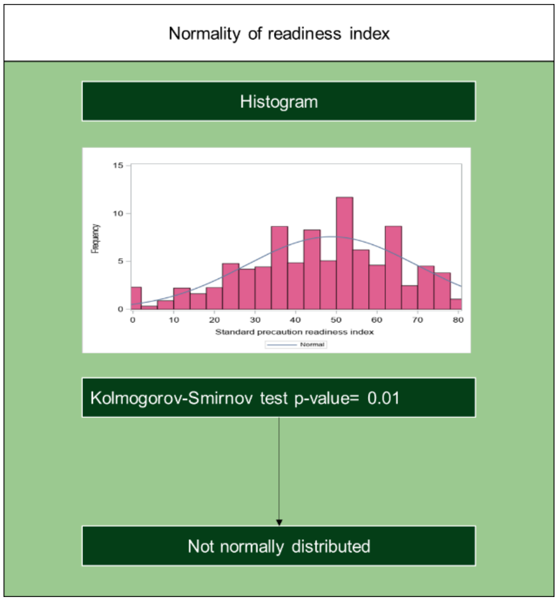

Figure S5: Normality of readiness index. The Kolmogorov-Smirnov test (p = 0.01) indicates a significant deviation from normality in the readiness score. However, we selected GEE because it estimated the mean and covariance structure without relying on a fully specified likelihood function. Existing studies show GEE computes point estimates under moderate skewness and non-normal

distribution data [6-8]. Additionally, standard errors obtained from GEE closely reflect empirical variability, supporting its suitability for analyzing correlated and non-normally distributed data [6-8].

Model Diagnosis

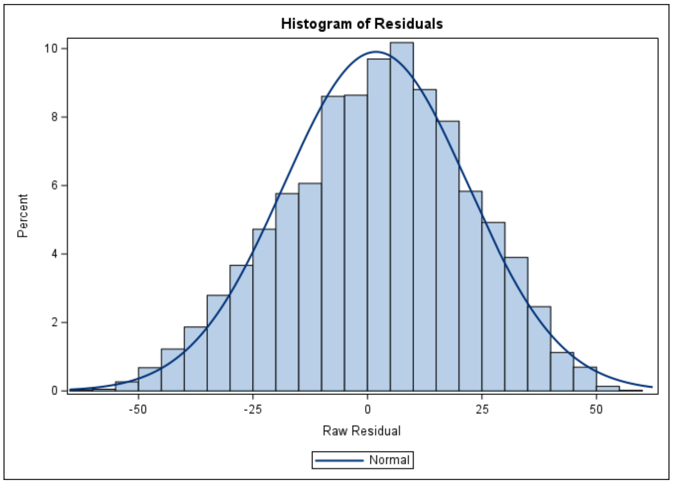

Figure S6: Histogram of Residuals. The histogram appears nearly bell-shaped with slight deviations from perfect normality, indicating that the residuals are approximately normally distributed.

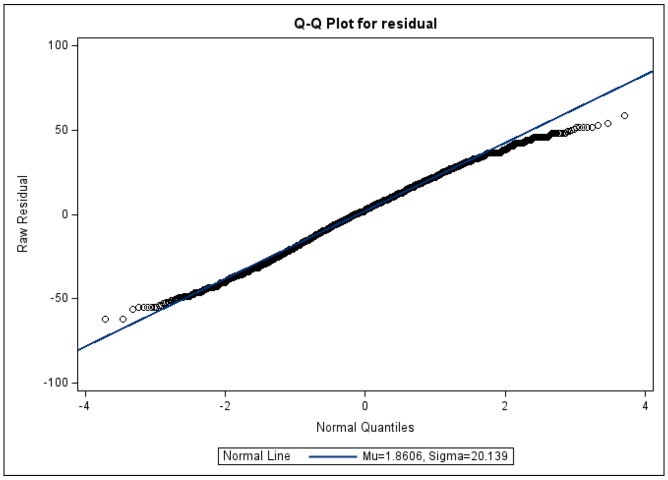

Figure S7: quantile-quantile plot. Since the residuals mostly align with the normal reference line, the assumption of normality of residuals is reasonably met. Slight deviations at the extremes may indicate the presence of outliers or slight skewness, but unless these are severe, they are unlikely to substantially affect model validity.

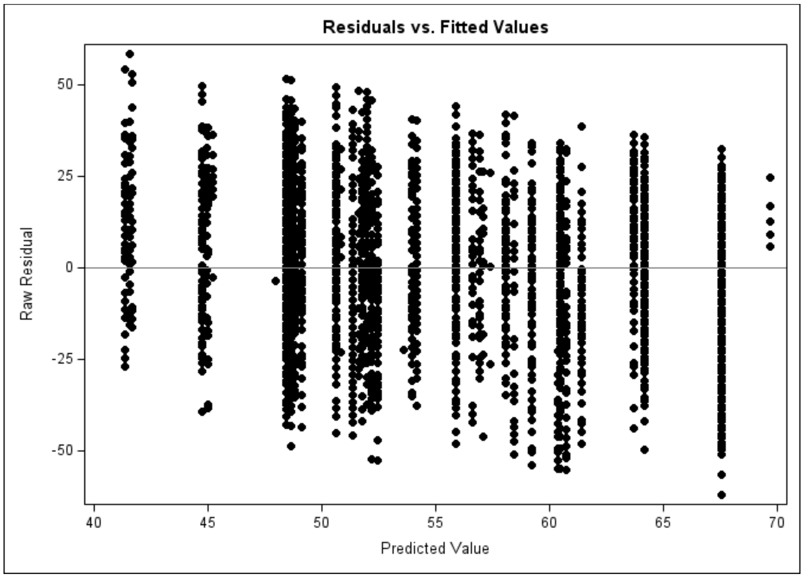

Figure S8: Residuals vs. Fitted values plot. The Residuals vs. Fitted Values plot shows that residuals are evenly spread around zero, supporting the assumption of constant variance (homoscedasticity).

While some variation in spread exists, there is no clear pattern, suggesting that the model's errors are stable. Also, the person correlation coefficient between residual and fitted values was 0.1, similar to the Spearman correlation coefficient, indicating a minimal degree of model misspecification.

Reference:

1 Huang FL. Analyzing cross-sectionally clustered data using generalized estimating equations. Journal of Educational and Behavioral Statistics. 2022;47:101-25.

2 Cui J, Qian G. Selection of working correlation structure and best model in GEE analyses of longitudinal data. Communications in statistics—Simulation and computation®. 2007;36:987-96.

3 Westgate PM, West BT. Tools for selecting working correlation structures when using weighted GEE to model longitudinal survey data. Journal of survey statistics and methodology. 2021;9:141-58.

4 Lee YL, Lim YMF, Law KB, Sivasampu S. Intra-cluster correlation coefficients in primary care patients with type 2 diabetes and hypertension. Trials. 2020;21:1-10.

5 Thompson DM, Fernald DH, Mold JW. Intraclass correlation coefficients typical of cluster-randomized studies: estimates from the Robert Wood Johnson Prescription for Health projects. The Annals of Family Medicine. 2012;10:235-40.

6 Song J, Barnhart HX, Lyles RH. A GEE approach for estimating correlation coefficients involving left-censored variables. Journal of Data Science. 2004;2:245-57.

7 Brobbey A. Classification Models for Multivariate Non-normal Repeated Measures Data. 2021.

8 Kabir A, Rashid MM, Hossain K, Khan A, Sikder SS, Gidding HF. Women's empowerment is associated with maternal nutrition and low birth weight: evidence from Bangladesh Demographic Health Survey. BMC women's health. 2020;20:1-12.
